# Expression of Four Autophagy-Related Genes Accurately Predicts the Prognosis of Gastrointestinal Cancer in Asian Patients

**DOI:** 10.1155/2021/7253633

**Published:** 2021-08-26

**Authors:** Hua Tang, Yinyin Liang, Shuyu Xu, Rong Xia, Jiemiao Shen, Yuxin Zhang, Xing Gong, Yue Min, Di Zhang, Tie Zhao, Shoulin Wang, Yi Zhang, Chao Wang

**Affiliations:** ^1^Department of General Surgery, Yueqing People's Hospital, 338 Qingyuan road, Yueqing, 325600 Zhejiang Province, China; ^2^Key Lab of Modern Toxicology of Ministry of Education, Center for Global Health, School of Public Health, Nanjing Medical University, 101 Longmian Avenue, Nanjing 211166, China; ^3^State Key Lab of Reproductive Medicine, Institute of Toxicology, Nanjing Medical University, 101 Longmian Avenue, Nanjing 211166, China; ^4^The First Clinical Medical College of Nanjing Medical University, Nanjing 211166, China; ^5^Pathological Department, Tongling People's Hospital, 468 Bijiashan Road, Tongling, Anhui Province 244000, China; ^6^Department of Colorectal Surgery, The First Affiliated Hospital of Nanjing Medical University, Nanjing 210000, China

## Abstract

Gastrointestinal (GI) cancers are among the most fatal diseases in the world. Numerous studies have demonstrated the relationship between autophagy and development of gastrointestinal cancers. However, whether autophagy-related genes can predict prognosis of GI cancers in individuals of Asian ancestry has not been defined. This study, evaluated the prognostic value of autophagy-related genes in gastrointestinal cancer. Expression profile of autophagy-related genes for 296 gastrointestinal cancer patients of Asian ancestry was downloaded from the TCGA database (TCGA-LIHC, TCGA-STAD, TCGA-ESCA, TCGA-PAAD, TCGA-COAD, TCGA-CHOL, and TCGA-READ). The prognostic value of the autophagy-related genes was evaluated using univariate Cox, LASSO, and multivariate Cox regression analyses. The risk score of the autophagy-related gene signature was calculated to assess its predictive prognostic value for GI cancers. Forty-seven differentially expressed autophagy-related genes, in Asian patients with gastrointestinal cancers, were identified. Of the 47 genes, 4 were associated with prognosis of GI cancer (SQSTM1, BIRC5, NRG3, and CXCR4). A prognostic model for GI cancer, based on the expression of the above 4 genes in the training set, showed that cancer patients were stratified into high-risk and low-risk groups (*P* < 0.05). The utility of the model for overall survival (OS) of GI cancer patients was consistent across the entire set, training set, and test set (entire set: *P* = 4.568 × 10^−4^; train set: *P* = 5.718 × 10^−3^; test set: *P* = 3.516 × 10^−2^). The sensitivity and specificity of the ROC curve of the above prognostic model in predicting the 5-year prognosis of GI cancer was satisfactory (entire set: 0.728; train set: 0.727; test set: 0.733). Analysis of clinical samples validated the overexpression of the 4 genes (SQSTM1, BIRC5, NRG3, and CXCR4) in tumor tissues relative to paired normal tissues, consistent with bioinformatic findings. Expression of the 4 autophagy-related genes (SQSTM1, BIRC5, NRG3, and CXCR4) can accurately predict the prognosis of gastrointestinal tumors in Asian patients.

## 1. Introduction

Digestive tract diseases are currently some of the most serious health problems worldwide. In the past two years, gastrointestinal (GI) cancers have caused significant mortalities. Gastric cancer, for instance, the fifth most common cancer, is the third leading cause of cancer-related deaths [1]. On the other hand, colorectal cancer is the fourth most fatal cancer, with nearly 900,000 deaths annually [2]. The majority of GI cancers relapse within five years of surgical resection. In 2019, the 5-year overall survival rate after gastric cancer in China was approximately 20% [3], whereas that of colorectal cancer was 60% [4]. Consequently, accurate markers for therapy response are required to improve the prognosis of GI cancer patients [5].

Autophagy selectively targets dysfunctional organelles, intracellular microbes, and proteins. Studies have demonstrated an etiological link between mutations in autophagy-related genes and human diseases [6]. Induction of autophagy recruits autophagy-related genes (ATGs) to the phagophore assembly site (PAS) to form the phagophore. The phagophore expands into a sphere around the cytosol and later matures into an autolysosome that contains phagocytosolic material. Aided by ATGs, autophagosomes fuse with the lysosome to form autolysomoses, which release the monomembranous particles that degrade the target materials [7]. This pathway is regulated by numerous molecules including core ATG protein, master cell growth regulator serine/threonine kinase mTOR, Beclin1, and antiapoptotic molecule BCL2 [8]. Several recent studies have uncovered new autophagy-related molecules, especially in tumors. Accumulating evidence shows that autophagy is a double-edged sword that can either promote or suppress tumor progression [9]. It is suggested that autophagy can inhibit early processes of tumorigenesis. In contrast, autophagy promotes growth of established tumors by allowing cancer cells to survive metabolic and therapeutic stress [10]. Recently, Nassour et al. reported that autophagy inhibits tumor growth and that loss of autophagy enhances cancer development [11]. Meanwhile, the reliance on autophagy for survival of many tumors implies that inhibiting autophagy is a potential treatment strategy against cancer. Although the role of autophagy in tumor treatment is still controversial, there is evidence that autophagy regulates resistance to tumor therapy, particularly GI tumors [12]. Thus, autophagy promotes progression of gastric cancer by participating in the development of chemotherapy resistance. Intestinal *Fusobacterium* infection promotes resistance to chemotherapy of colorectal cancer by activating innate immunity and autophageal pathways [13]. Therefore, identifying autophagy-related genes may unravel the genetic prognostic indicators for GI cancers. In addition, it may form a basis upon which novel drugs that address the different patient responses to chemotherapy can be developed.

In the past two years, many studies have provided new evidence for tumor therapy response using prognostic prediction models of autophagy-related genes. For instance, an independent four gene autophagy-related signature was revealed to accurately predict the prognosis of glioblastoma multiforme (GBM) [14]. In a related study, the autophagy-related gene signature was reported to be a promising prognostic molecular biomarker for prostate cancer (PCa) [15]. Elsewhere, a five autophagy-related gene model that independently predicts the OS of endometrial cancer (EC) patients has been developed [16]. However, autophagy-related genes for the prognosis of gastrointestinal cancer in Asian patients remain to be unraveled.

The present study analyzed data in the TCGA repository to uncover the autophagy-related genes associated with clinical characteristics and prognosis of GI cancer in Asian patients. In addition, a gene expression model stratifying patients into high-risk or low-risk groups, based on the expression profiles of ARGs, was built. The sensitivity and specificity of this model were validated using several patient datasets.

## 2. Materials and Methods

### 2.1. mRNA Expression Data for Gastrointestinal Cancer in Asian Patients

The mRNA expression profiles and the corresponding clinical data of 296 GI cancer patients of Asian ancestry were obtained from the TCGA database (http://cancergenome.nih.gov/). Selection criteria were limited to the mRNA expression data from the TCGA database. Data was included based on the following: (1) the data belongs to Asian patients with GI cancer; (2) have basic clinical information, including overall survival (OS), survival status, age, gender, clinical grade, and stage. This included data was for 158 LIHC, 74 STAD, 38 ESCA, 11 PAAD, 11 COAD, 3 CHOL, and 1 READ patient. Details of clinical characteristics of the patients are summarized in Figure S1. Gene set enrichment analysis of the differently expressed autophagy-related genes was performed using the Molecular Signatures Database V.7.0 (MSigDB), accessible at https://software.broadinstitute.org/gsea/msigdb/index.jsp.

### 2.2. Identification of Differently Expressed ARGs

The differentially expressed ARGs between GI tumor and matched nontumor tissues were identified using the limma package in R software. Gene expression of more than onefold change and FDR < 0.05 was considered significant. Further, the biological functions and pathways regulated by the differently expressed genes (DEGs) were analyzed based on Gene Ontology (GO) and Kyoto Encyclopedia of Genes and Genomes (KEGG) databases (http://amp.pharm.mssm.edu/Enrichr/).

### 2.3. Prognostic Signature Construction Based on ARGs

The expression of ARGs was first normalized based on [log2 (data+1)] before further analysis. The GI cancer patients were randomly separated into the training and test in the ratio of 6/4. Thereafter, univariate, LASSO, and multivariate Cox regression analyses were performed to identify the significance of the DEGs between GI cancer and paired normal genes. Time-dependent receiver-operating characteristic (ROC) analysis was used to estimate the risk score of a patient. The accuracy of the model was validated using the curve (AUC). Patients were stratified into high-risk and low-risk groups based on the median risk score, calculated as follows: Risk score = [(expression of gene 1 × *β*1gene 1) + (expression of gene 2 × *β*2 gene 2)+⋯(expression of gene n × *βn* gene *n*)], where *β* represents the regression coefficient of each mRNA. Univariate and multivariate Cox regression analyses were performed to assess the prognostic value and independence of the model. Clinicopathologic characteristics of patients downloaded from the TCGA database are shown in Figure S1. The tumor type, age, stage, gender, and vital status were used as covariates. The model was validated using a test set as well as the entire set.

### 2.4. Gene Set Enrichment Analysis (GSEA)

The statistical significance of the DEGs was validated based on GSEA analysis using the GSEA software V.4.0.1 and “h.all.v7.1.symbols.gmt” (http://www.broadinstitute.org/gsea). *P* value < 0.05 and false discovery rate (FDR) < 0.25 were considered statistically significant.

### 2.5. Sample Collection and Validation of ARG Expression

The protocol for this research was approved by the Institutional Review Board of Nanjing Medical University and the Ethical Committee of the Tongling People's Hospital (ethical review no. 2019-008). Twenty-eight paired GI tumor and adjacent nontumor tissues were extracted from patients attending the Tongling People's Hospital from 2018 to 2019. All patients consented to participate in the study. The patients had not received chemotherapy or radiotherapy prior to surgery. The tissue pairs were extracted from 28 cancer patients, including 8 COAD, 5 READ, and 15 STAD patients. Details of the patients are summarized in Supplementary material Table S1. The tissue samples were rapidly frozen and stored in liquid nitrogen pending RNA extraction. Total RNA was extracted and reverse transcribed as previously described [17]. The primer sequences were as follows: GAPDH; CCTTCCGTGTCCCCACT and GCCTGCTTCACCACCTTC, BIRC5; GGACCACCGCATCTCTAC and CCAAGTCTGGCTCGTTCT, CXCR4; AATCTTCCTGCCCACCA and CTTGTCCGTCATGCTTCTC, NRG3; CGCTACCTCCTCCTACCTT and AGTTTCTGGTGTGGTGGTG, SQSTM1; GCAGCCCAGCACATAGC and CTTCTCAGTCCCAGCAGGA. For all the genes, the forward primers are listed first. All experiments were repeated three times.

### 2.6. Statistical Analysis

The identification of survival ARGs and the construction of the risk score model were performed using univariate and multivariate Cox regression analyses, respectively. The survival rates between high-risk and low-risk groups were evaluated using the log-rank test. The survival ROC package in R software was used to plot ROC and AUC. The relative group risks were assessed based on hazard ratios (HRs) and 95% confidence interval (CI). The survival curves for the risk groups, based on clinic-pathologic characteristics and model genes, were plotted using the Kaplan-Meier method. All statistical analyses were performed using R software V. 3.6.3 and GraphPad Prism 7 software. *P* < 0.05 was considered statistically significant.

## 3. Results

### 3.1. Identification of Differentially Expressed ARGs

**Figure 1:**
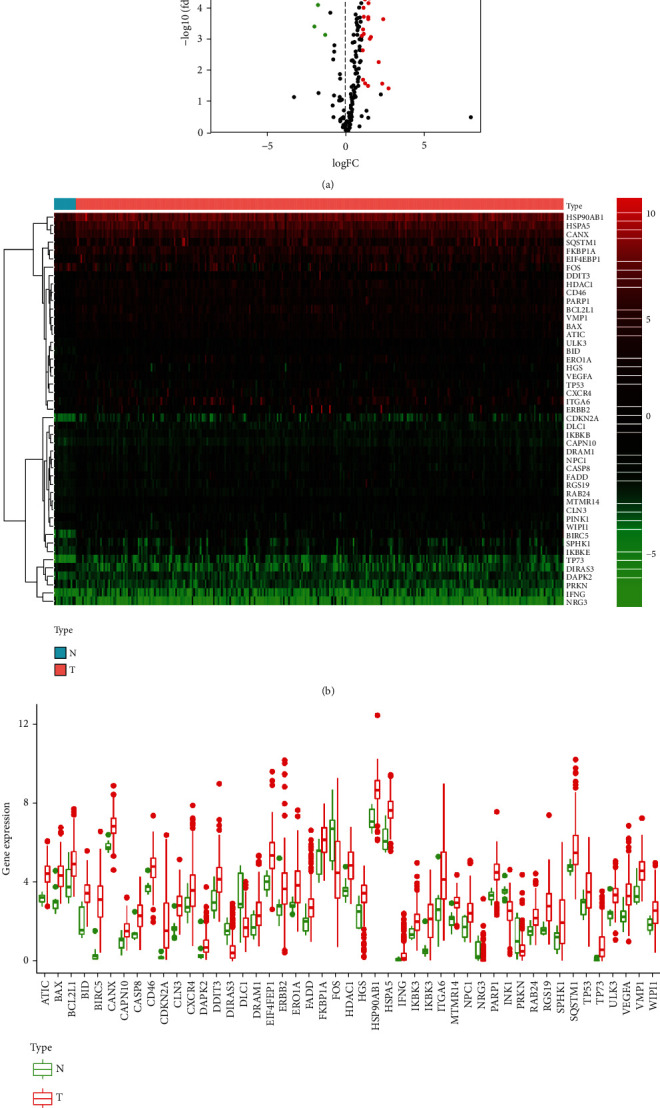
Differentially expressed autophagy-related genes (ARGs): (a) volcano plot and (b) heatmap for differently expressed ARGs between cancer and normal tissues. Red dots represent high expression, whereas green represents low expression. (c) The expression patterns of 47 ARGs in tumor and paired nontumor tissues. Each dot represents expression of a sample; red dots represent tumor tissues, whereas the green dots represent normal tissues.

The mRNA expression profiles and the corresponding clinical data of 296 GI cancer patients of Asian ancestry were obtained from the TCGA dataset. With ∣log2 (Fold Change) | >0 and FDR < 0.05, 47 differentially expressed ARGs were revealed. Of the 47 ARGs, 41 were upregulated and 6 were downregulated. The DEGs between tumor and adjacent normal tissues are shown in the volcano plot (Figure 1(a)) and heatmap plot (Figure 1(b)).  Figure 1(c) shows the box plots displaying the expression patterns of the differentially expressed ARGs, including 41 upregulated (ATIC, BAX, BCL2L1, BID, BIRC5, CANX, CAPN10, CASP8, CD46, CDKN2A, CLN3, CXCR4, DAPK2, DDIT3, DRAM1, EIF4EBP1, ERBB2, ERO1A, FADD, FKBP1A, HDAC1, HSP90AB1, HSPA5, IFNG, IKBKB, IKBKE, ITGA6, MTMR14, NPC1, NRG3, PARP1, RAB24, RGS19, SPHK1, SQSTM1, TP53, TP73, ULK3, VEGFA, VMP1, and WIPI1) and 6 downregulated (DIRAS3, DLC1, FOS, HGS, PINK1, and PRKN) genes. The Gene Ontology (GO) enrichment analyses revealed that the DEGs participated in autophageal processes, macroautophagy, neuron death, and intrinsic apoptotic signaling pathway. On the other hand, KEGG revealed that the DEGs regulated apoptosis, measles, platinum drug resistance, p53 signaling, Kaposi sarcoma-associated herpesvirus infection, and IL-17 signaling pathways (Figure S2).

### 3.2. The Risk Score Model for the Entire Set

**Figure 2:**
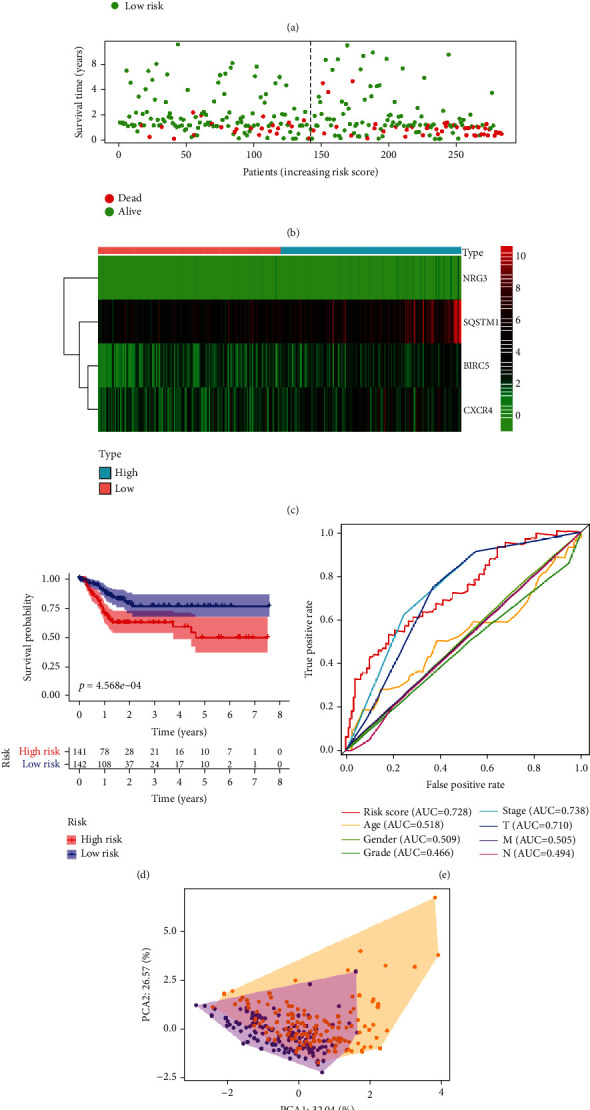
Prognostic value of the autophagy-related genes based on the entire set analysis: (a) the risk scores for low- and high-risk group patients. (b) The overall survival of the two risk group patients. (c) Heatmap for the expression of ARGs. (d) Kaplan-Meier survival analysis for the entire set. (e) Time-dependent ROC curve for the risk score in predicting the overall survival of GI cancer patients. (f) Principal component analysis plots for the entire set.

**Figure 3:**
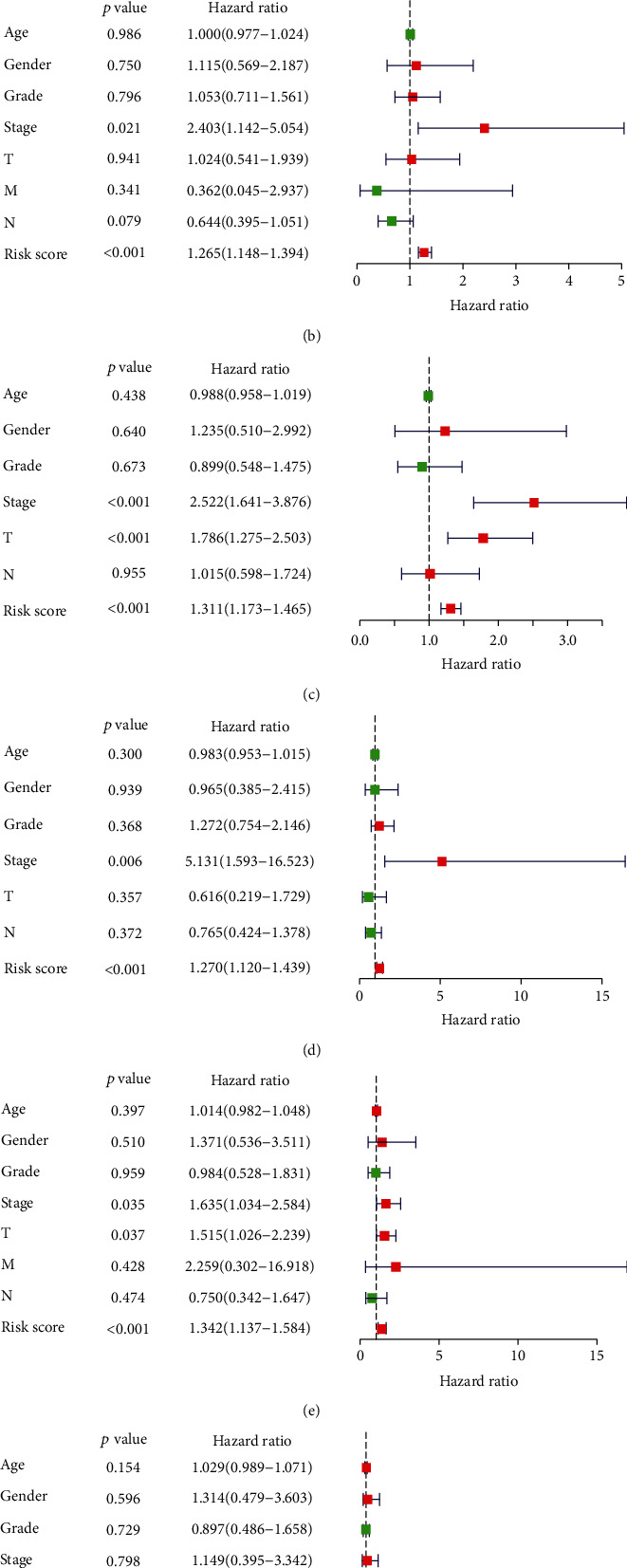
Univariate and multivariate logistic regression analyses for the prognostic value of the GI cancer linked ARGs: univariate (a, c, e) and multivariate (b, d, f) regression analyses for the prognostic value of ARGs in combination with clinicopathologic factors in GI cancers based on the entire set data (a, b), train set (c, d), and (e, f) the test set.

The risk score model integrated data for ARG expression profiles and clinical characteristics. The univariate, LASSO, and multivariate Cox regression analyses for the entire set (Figure S3) revealed that SQSTM1, BIRC5, CXCR4, and NRG3 were the top 4 most significant differently expressed ARGs. The prognosis scores were calculated as follows: risk score = (0.441279 × expression of SQSTM1 + 0.601246 × expression of NRG3 + 0.415784 × expression of BIRC5 + 0.241966 × expression of CXCR4) (Table 1). The expression of the above genes was negatively related to the OS of GI cancer patients. Based on the median risk score (Figures 2(a)–2(c)), patients were stratified into high-risk and low-risk groups. The survival probability of the low-risk group was significantly higher than that of high-risk group individuals (*P* = 4.568 × 10^−4^) (Figure 2(d)). The time-dependent AUC based on ROC analysis was 0.728 for overall risk, 0.518 for age, 0.509 for gender, 0.466 for tumor grade, 0.738 for tumor stage, 0.710 for tumor (T), 0.505 for metastasis (M), and 0.494 for node (N) (Figure 2(e)). Principal component analysis revealed a different pattern of high and low risk according to five autophagy-related genes in this training set (Figure 2(f)). Univariate and multivariate Cox regression analyses revealed that the model was an accurate independent predictor of GI cancer prognosis of Asian patients (Figures 3(a) and 3(b)).

### 3.3. Validation of the Risk Model Using the Training Set

**Figure 4:**
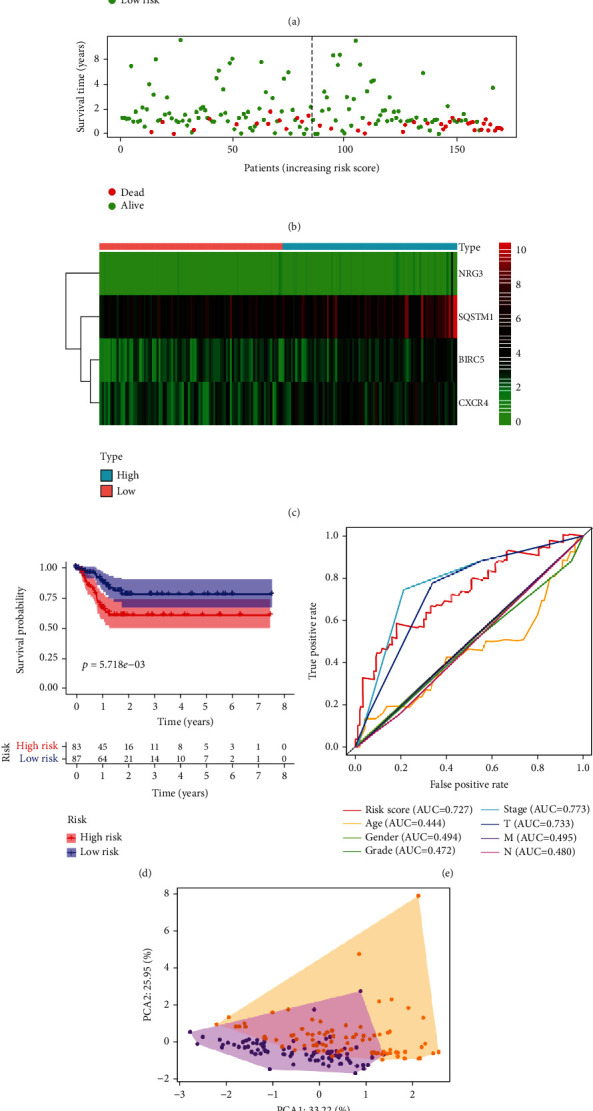
Prognostic value analysis of ARGs based on the training set: (a) the risk scores for patients in low- and high-risk groups. (b) The overall survival of patients in low- and high-risk groups. (c) Heatmap for the expression of ARGs in normal and paired GI cancer tissues. (d) Kaplan-Meier survival analysis for the training set. (e) Time-dependent ROC for the prediction of overall survival of GI cancer patients. (f) Principal component analysis plots for the training set.

The accuracy and utility of our model was validated using the training and test sets, derived from the original set. The risk scores and survival status of patients in high- or low-risk score groups in the training set are shown in Figures 4(a)–4(c). As shown in Figure 4(d), the survival time of the low-risk group was significantly longer than that of high-risk group individuals (*P* = 5.718 × 10^−3^). This was consistent with bioinformatic analyses. The AUC for risk score, age, gender, grade, stage, T, M, and N were 0.727, 0.444, 0.494, 0.472, 0.773, 0.733, 0.495, and 0.480, respectively (Figure 4(e)). Principal component analysis displayed a different pattern of high-risk and low-risk groups based on the gene at the test set (Figure 4(f)). Univariate and multivariate Cox regression analyses revealed that the model could accurately and independently predict the prognosis of GI cancer (Figures 3(c) and 3(d)).

### 3.4. Validation of the Risk Model Using the Test Set

**Figure 5:**
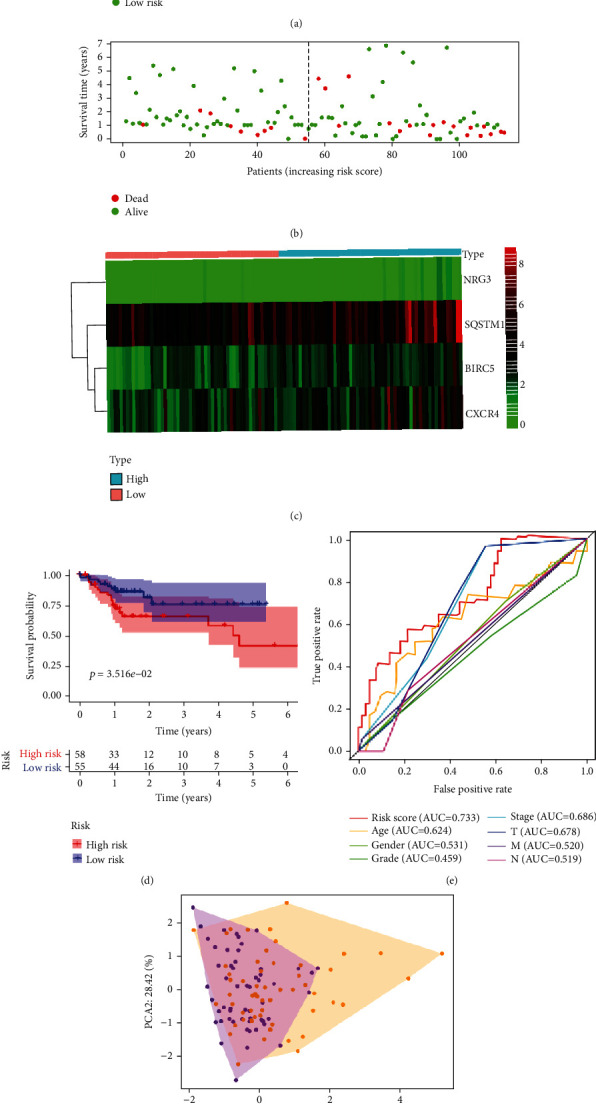
Prognostic analysis of the test set. (a) The risk scores for low- and high-risk group patients. (b) The overall survival of the low- and high-risk group patients. (c) Heatmap for the expression of ARGs associated with GI cancers. (d) Kaplan-Meier survival analysis for the test set. (e) Risk score-based time-dependent ROC curve for prediction of overall survival of GI cancer patients. (f) Principal component analysis plots for the test set.

Patients in the test set were stratified into high-risk and low-risk groups based on the median risk score. The risk scores and survival status of the patients are shown in Figures 5(a)–5(c). As shown in Figure 5(d), low-risk group patients displayed significantly longer survival time than their counterparts in the high-risk group (*P* = 3.516 × 10^−2^), consistent with whole dataset analyses. The AUC based on ROC for overall risk score, age, gender, grade, stage, T, M, and N were 0.733, 0.642, 0.531, 0.459, 0.686, 0.678, 0.520, and 0.519, respectively (Figure 5(e)). Principal component analysis displayed a different pattern of high-risk and low-risk groups based on the gene at the test set (Figure 5(f)). Just like for the entire dataset, univariate and multivariate Cox regression analyses validated the accuracy and independency of the prognostic model for GI cancer, further demonstrating the prognostic predictive value of the model among Asian patients (Figures 3(e) and 3(f)).

### 3.5. Relationship between the Risk Score and Clinicopathologic Characteristics

**Figure 6:**
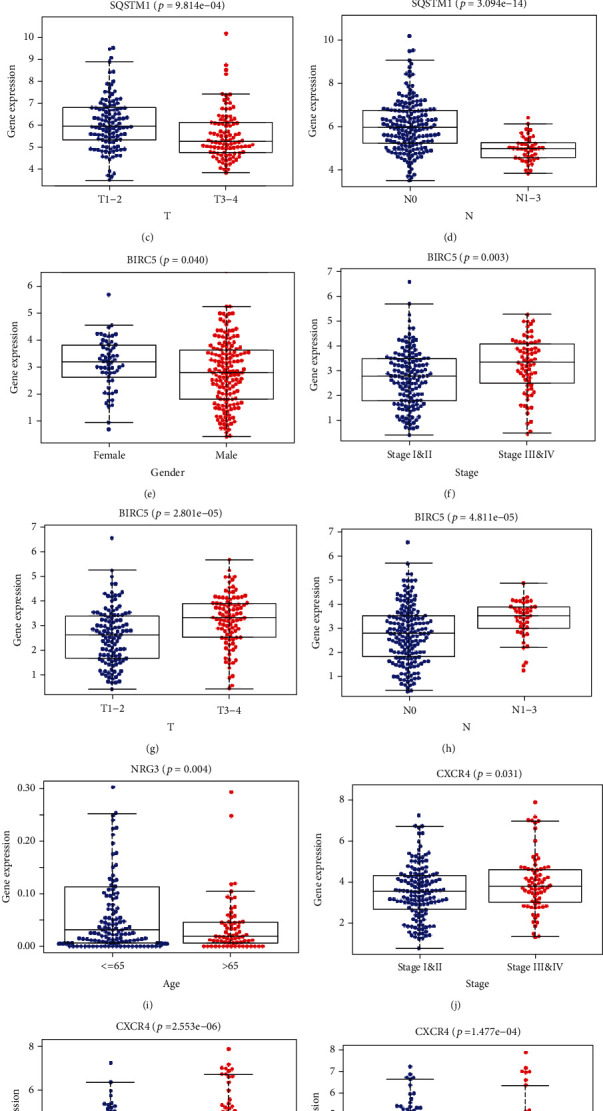
Clinical relevance of risk scores for ARGs in Asian GI cancer patients: relationship of risk score (a) or expression of SQSTM1 (b–d), BIRC5 (e–h), NRG3 (i), and CXCR4 genes (j–l) and prognosis of GI cancer.

The patients were also stratified into two groups along gender, age, stage, T, M, and N. The box plot for the relationship between the risk score and the expression profile of ARGs is shown in Figure 6. Risk scores for groups N1-3 were higher than that of N0 (*P* = 0.011) (Figure 6(a)). The SQSTM1 gene was overexpressed in males, T1-2, and N0 (Figures 6(b)–6(d)). The expression of BIRC5 was higher in females, in stage 3 and 4 tumors, T3-4, and N1-3 (Figures 6(e)–6(h)), whereas the expression of NRG3 was higher in patients under 65 years old (Figure 6(i)). The CXCR4 was overexpressed in cancer stages 3 and 4, T3-4, and N1-3 (Figures 6(j)–6(l)). The GSEA and GO analysis, based on risk score group, revealed that the DEGs regulated the “ubiquitin like protein ligase binding,” “RNA splicing via transesterification reactions,” “protein folding,” and “the chaperone mediated protein folding pathways.” The GSEA and KEGG analysis revealed that the genes participated in “spliceosome,” “RNA degradation,” “pyrimidine metabolism,” “purine metabolism,” and “natural killer cell-meditated cytotoxicity” (Figure S4).

### 3.6. Nomogram and Its Clinical Utility

**Figure 7:**
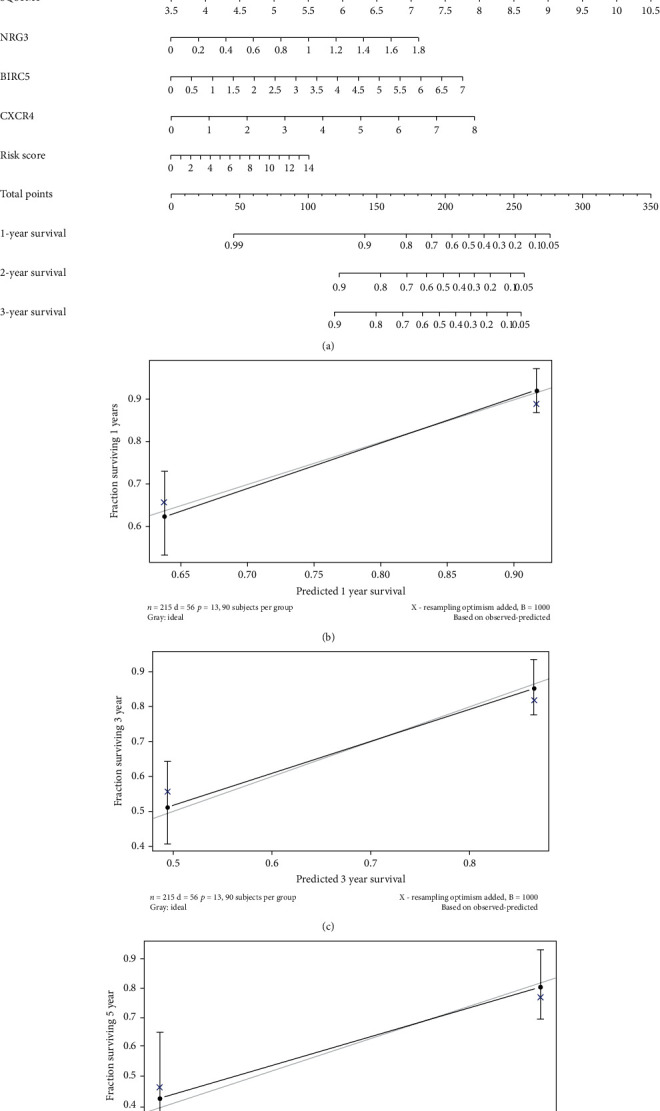
The novel nomogram for the prediction of overall survival of GI cancer patients based on nine independent prognostic factors: (a) the 1-, 3-, or 5-year prediction of OS of GI cancer patients in the entire set. (b–d) The calibration plots for the 1-, 3-, or 5-year OS prediction value of the nine-factor model.

**Figure 8:**
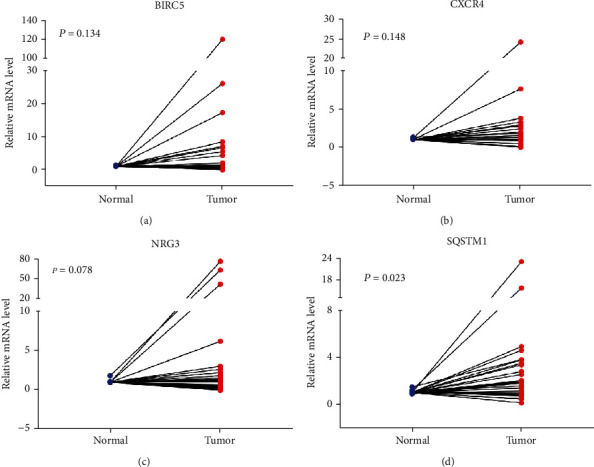
The mRNA expression level of four ARGs between normal and tumor tissues: (a) BIRC5, (b) CXCR4, (c) NRG3, and (d) SQSTM1.

The nomogram for the Asian GI cancer patients incorporated the risk scores and clinical factors (Figure 7(a)). The 1-year, 3-year, and 5-year survival prognosis prediction values were satisfactory (Figures 7(b)–7(d)). The qRT-PCR validated the expression of overexpressed mRNAs of the four predicted ARGs (BIRC5, CXCR4, NRG3, and SQSTM1) in clinical tissue samples (Figure 8).

## 4. Discussion

Gastrointestinal (GI) tumors are one of the most serious health complications in the world. They mainly include seven major cancers: liver hepatocellular carcinoma, stomach adenocarcinoma, esophageal carcinoma, pancreatic adenocarcinoma, colon adenocarcinoma, cholangiocarcinoma, and rectum adenocarcinoma [18]. Understanding the molecular markers that predict prognosis of GI tumors can guide clinical management of patients with these cancers. Several risk factors have been linked to each of the cancers of the GI. For instance, suggested risk factors for stomach adenocarcinoma include diet, lifestyle, genetics, treatment, and medical conditions, infection with certain bacteria and viruses, demographic characteristics, occupational exposure, and ionizing radiation [19]. Other studies have implicated genetic and environmental factors like obesity, poor diets, and alcohol drinking in the development of colorectal cancer [20]. However, given that most risk factors can only be determined after extensive surgery, this does not offer optimum solution to management of GI cancers. In view of this, minimally invasive alternatives in management of GI cancers are particularly necessary. Overall, reliable molecular biomarkers for predicting the prognosis of GI tumors are important in monitoring individual patient response to therapies.

Autophagy is a highly conservative catabolic energy producing process [21]. Recent studies have demonstrated the key role autophagy plays in multiple tumors, including GI types. Autophagy influences tumor metastasis, EMT, apoptosis, and drug resistance [22]. In gastric cancer, the expression pattern of Beclin1, LC3, and P62/SQSTM1, which are autophagy-related genes, predicts the prognosis of the tumor [23–25]. Another study has also identified 4 genes (GRID2, ATG4D, GABARAPL2, and CXCR4) as a potential prognostic marker for predicting the prognosis of GC patients [26]. Separate evidence shows that the expression of ATG5 and ATG7, autophagy-related proteins, influences the prognosis of colorectal cancer [27]. Overall, autophagy proteins can suppress or promote progression of tumors [28]. Indeed, several studies show that absence of autophagy is related to worse clinicopathological properties and adverse outcomes of HCC, implying that autophagy can inhibit development and progression of tumors [29]. However, whether autophagy has a prognostic role in GI tumors has not been validated.

In this study, we used TCGA data to identify ARGs and their associated pathways. Overall, we identified 4 main ARGs (SQSTM1, NRG3, BIRC5, and CXCR4) closely related to the overall prognosis of GI. The SQSTM1 is a multifunctional stress-inducible scaffold protein that regulates numerous cellular processes [30] such as activation of the nuclear factor kappa-8 signaling pathway. It also links polyubiquitinated cargo and autophagy [31]. Besides tumors, the protein is critical in several other disease types, including neurodegenerative [32], and cardiometabolic diseases [33], melanomas [34] as well as breast [35] and lung cancers [36]. Expression of SQSTM1 has been implicated in the development of GBM samples. In particular, p62 expression inversely correlates with that of GSK-3*β* in human GBM tissues. The expression of the two markers accurately predicted the prognosis of GBM [37]. Generally, SQSTM1 is downregulated in normal gastric tissues. However, overexpression of SQSTM1 in gastric cancer tissue is associated with poor overall survival [38]. High SQSTM1 levels continuously activate Nrf2 and its downstream target genes, which independently promote growth of liver cancer cells during the early stages of the disease [39]. The NRG3, in the larger neuregulin gene family, induces proliferation, migration, differentiation, and survival or apoptosis of cancer cells. By acting on ErbB4, NRG3 promotes the development of Hirschsprung disease (HSCR) [40]. In combination with NRG3, Williams-Beuren syndrome transcription (WSTF), a nonsecretory protein, activates oncogenesis of colon tumors [41]. BIRC5 is a member of the inhibitor of apoptosis genes. It encodes negative regulatory proteins that prevent apoptotic cell death. Also known as survivin, BIRC5 is a well-known cancer treatment target [42]. Overexpression of BIRC5 proteins in various cancers is associated with poor survival [43]. In particular, expression of BIRC5 negatively correlates with that of ATG7, but positivity correlates with that of SQSTM1 [44]. Overexpression of BIRC5 has been linked with the development and progression of esophageal, liver, colon, and gastric cancers [45]. The CXCR4 encodes a CXC chemokine receptor specific for stromal cell-derived factor-1, which regulates normal or abnormal biological processes and participates in numerous carcinogenesis pathways [46]. The combined expression of CXCR12 and CXCR4 activates G protein signal kinase, promoting the development of gastrointestinal tumors [47]. Also, CXCR4 has been shown to influence the overall survival of patients with GI cancer. Overexpression of high CXCR4 confers poor prognosis of oesophageal, gastric, and colorectal cancers [48]. Overall, SQSTM1, NRG3, BIRC5, and CXCR4 genes participate in the development or progression of GI cancer, consistent with the current study's findings.

An accurate prognosis prediction model was constructed using the autophagy-related genes identified from the TCGA database. Based on the model, patients can be stratified into low- and high-risk groups along age, grade, clinical stage, and histological type. The accuracy of the model in prognosis prediction was validated using the training and test sets. The four model genes are overexpressed in tumor tissues, relative to normal tissues. This highlights the clinical application of these genes. Although this study provides interesting findings, there are some limitations worth mentioning. First, the data used in this study is relatively small, which casts doubt on the credibility of the model proposed. Second, some prognostic-related factors like tumor size, lymph node metastasis, and immune infiltration were not investigated. Therefore, prospect cohort studies with large sample size are needed to further confirm the clinical utility and biological function of our model. Overall, when combined with clinical characteristics, the expression of four autophagy-related genes (SQSTM1, BIRC5, NRG3, and CXCR4) can accurately predict the overall survival of Asian patients with GI cancer.

## Figures and Tables

**Table 1:** The information of four prognostic mRNAs weighted by its multivariable LASSO regression coefficient, which is importantly associated with overall survival in Asian patients with gastrointestinal cancer.

mRNA	Ensemble ID	Location	Risk coefficient	HR (95% CI)	*P* value
SQSTM1	ENSG00000161011	Chromosome 5: 179, 806, 398-179, 838, 078	0.441279	1.554695 (1.288022-1.87658)	4.30E-06
NRG3	ENSG00000185737	Chromosome 10: 81, 875, 194-82, 987, 179	0.601246	1.82439 (1.133882-2.935403)	0.013221
BIRC5	ENSG00000089685	Chromosome 17: 78, 214, 186-78, 225, 636	0.415784	1.515558 (1.232382-1.863803)	8.15E-05
CXCR4	ENSG00000121966	Chromosome 2: 136, 114, 349-136, 118, 149	0.241966	1.273751 (1.058673-1.532524)	0.010342

## Data Availability

The data sets used and/or analyzed during the current study are publicly available data from The Cancer Genome Atlas (TCGA). The figures and materials supporting the conclusions of this article are included within the article.

## Abbreviations

TCGA: The Cancer Genome Atlas
LIHC: Liver hepatocellular carcinoma
STAD: Stomach adenocarcinoma
ESCA: Esophageal carcinoma
PAAD: Pancreatic adenocarcinoma
COAD: Colon adenocarcinoma
CHOL: Cholangiocarcinoma
READ: Rectum adenocarcinoma
GI cancer: Gastrointestinal cancer
SQSTM1: Sequestosome 1
BIRC5: Baculoviral IAP repeat containing 5
NRG3: Neuregulin 3
CXCR4: C-X-C motif chemokine receptor 4
ROC curve: Receiver-operating characteristic curve
OS: Overall survival
ARGs: Autophagy-related genes
GBM: Glioblastoma
PCa: Prostate cancer
EC: Endometrial cancer
GO: Gene Ontology
KEGG: Kyoto Encyclopedia of Gene and Genomes
AUC: Area under the curve
GSEA: Gene set enrichment analysis
FDR: False discovery rate
HRs: Hazard ratios
CIs: Confidence intervals.

## Supplementary Materials

Supplementary Materials Table S1: the characteristics of tumor samples used in this study. Figure S1: clinical pathological parameters of Asian patients with gastrointestinal cancer in this research. Figure S2: GO and KEGG pathway enrichment analyses of the DEGs in GI cancers. (A) GO and (B) KEGG. Figure S3: prognosis-related ARGs based on LASSO regression analysis. (A) LASSO coefficient for the ARGs associated with the overall survival of GI cancer. (B) Plots of the cross-validation error rates. Figure S4: significant pathways in high- and low-risk groups of GI cancer patients: (A) GO based on GSEA; (B) KEGG based on GSEA.
