# Supplementary material for: Expression of Four Autophagy-Related Genes Accurately Predicts the Prognosis of Gastrointestinal Cancer in Asian Patients
Source: Dis Markers. 2021 Aug 26;2021:7253633. doi: 10.1155/2021/7253633 (PMC8413069; doi:10.1155/2021/7253633)

A

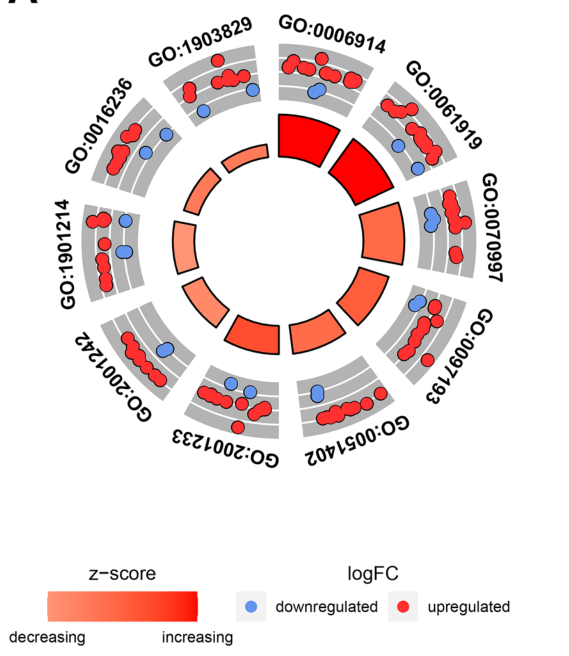

ID	Description
GO:0006914	autophagy
GO:0061919	process utilizing autophagic mechanism
GO:0070997	neuron death
GO:0097193	intrinsic apoptotic signaling pathway
GO:0051402	neuron apoptotic process
GO:2001233	regulation of apoptotic signaling pathway
GO:2001242	regulation of intrinsic apoptotic signaling pathway
GO:1901214	regulation of neuron death
GO:0016236	macroautophagy
GO:1903829	positive regulation of cellular protein localization

B

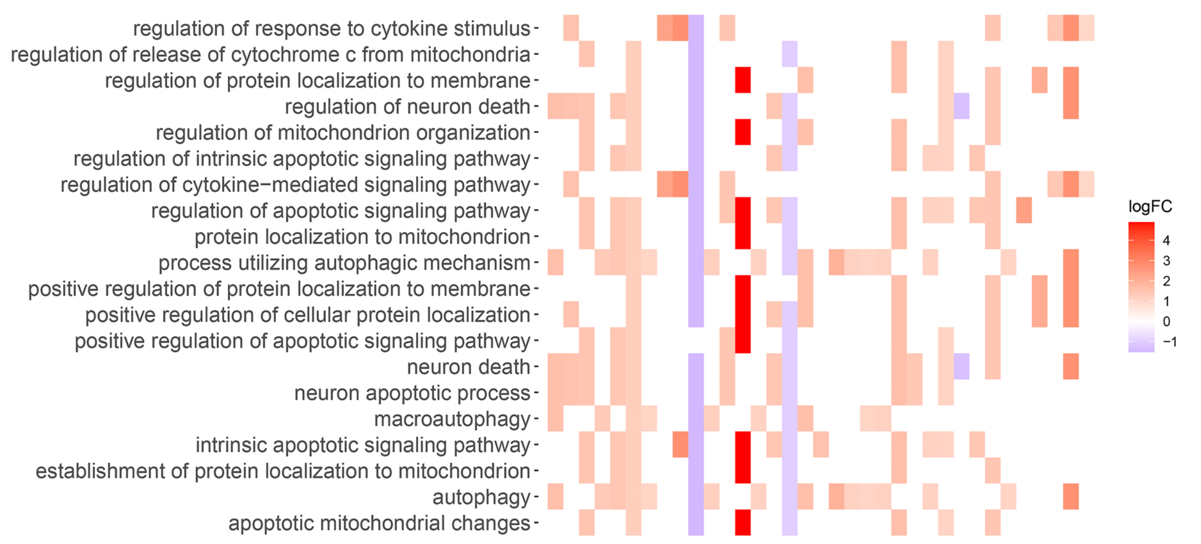

C

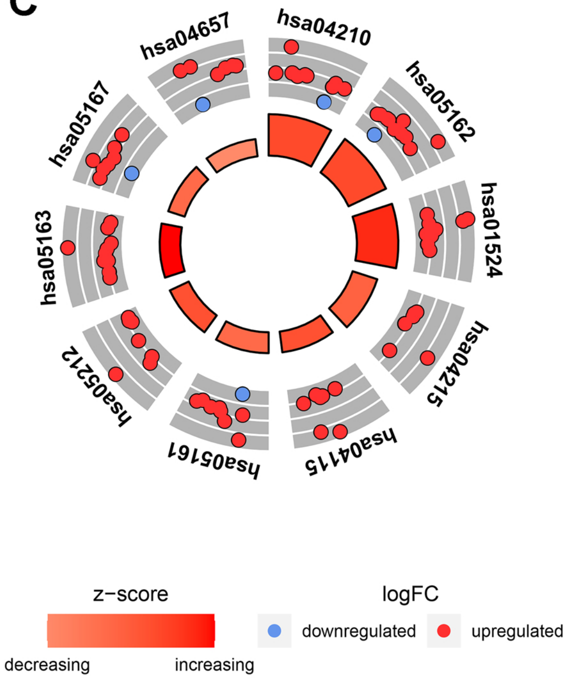

ID	Description
hsa04210	Apoptosis
hsa05162	Measles
hsa01524	Platinum drug resistance
hsa04215	Apoptosis − multiple species
hsa04115	p53 signaling pathway
hsa05161	Hepatitis B
hsa05212	Pancreatic cancer
hsa05163	Human cytomegalovirus infection
hsa05167	Kaposi sarcoma−associated herpesvirus infectior
hsa04657	IL−17 signaling pathway

D

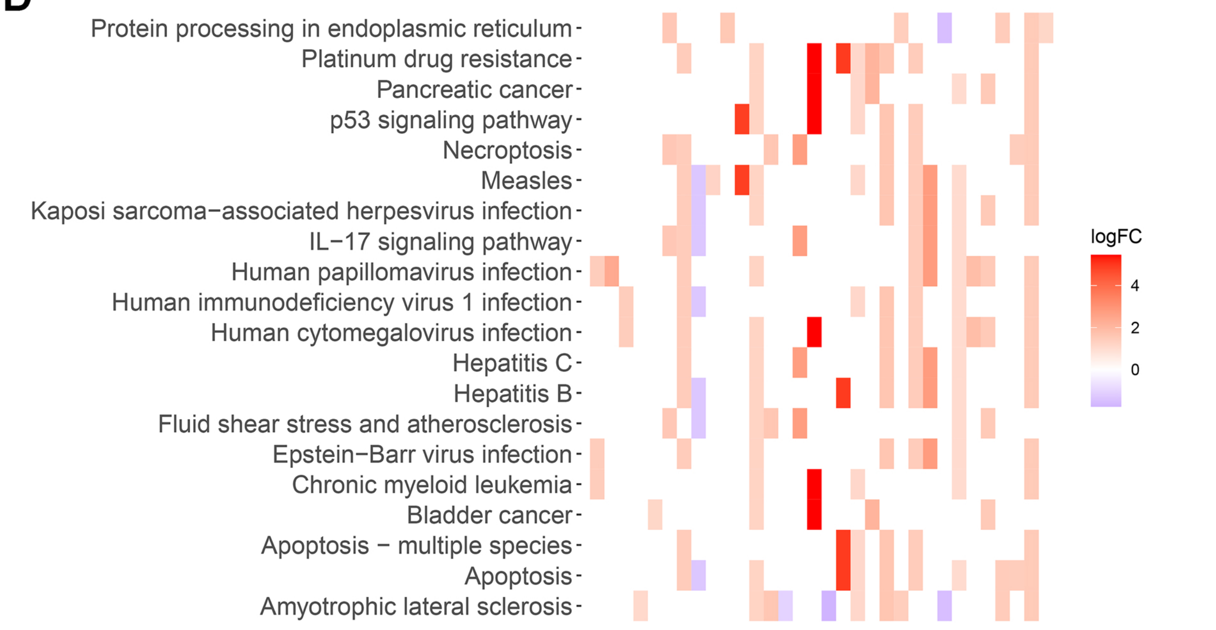

Supplement: Supplementary Materials — Table S1: the characteristics of tumor samples used in this study. Figure S1: clinical pathological parameters of Asian patients with gastrointestinal cancer in this research. Figure S2: GO and KEGG pathway enrichment analyses of the DEGs in GI cancers. (A) GO and (B) KEGG. Figure S3: prognosis-related ARGs based on LASSO regression analysis. (A) LASSO coefficient for the ARGs associated with the overall survival of GI cancer. (B) Plots of the cross-validation error rates. Figure S4: significant pathways in high- and low-risk groups of GI cancer patients: (A) GO based on GSEA; (B) KEGG based on GSEA. [file 7253633.f1.zip › 7253633.f1/Supplementary material Figure S2.pdf]
