# Supplementary material for: Expression of Four Autophagy-Related Genes Accurately Predicts the Prognosis of Gastrointestinal Cancer in Asian Patients
Source: Dis Markers. 2021 Aug 26;2021:7253633. doi: 10.1155/2021/7253633 (PMC8413069; doi:10.1155/2021/7253633)
Supplement: Supplementary Materials — Table S1: the characteristics of tumor samples used in this study. Figure S1: clinical pathological parameters of Asian patients with gastrointestinal cancer in this research. Figure S2: GO and KEGG pathway enrichment analyses of the DEGs in GI cancers. (A) GO and (B) KEGG. Figure S3: prognosis-related ARGs based on LASSO regression analysis. (A) LASSO coefficient for the ARGs associated with the overall survival of GI cancer. (B) Plots of the cross-validation error rates. Figure S4: significant pathways in high- and low-risk groups of GI cancer patients: (A) GO based on GSEA; (B) KEGG based on GSEA. [file 7253633.f1.zip › 7253633.f1/Supplementary material Table S1.pdf]

No.	Gender	Age	Tumor	Grade
1	female	79	Colon cancer	IIb
2	male	59	Colon cancer	IIIb
3	male	64	Colon cancer	IIIc
4	female	68	Colon cancer	IIIc
5	male	74	Colon cancer	IIb
6	male	58	Colon cancer	IIIb
7	female	73	Colon cancer	Ib
8	male	66	Colon cancer	IIa
9	male	49	Gastric cancer	IIIb
10	male	63	Gastric cancer	Ib
11	male	74	Gastric cancer	IIIa
12	male	63	Gastric cancer	IIb
13	male	69	Gastric cancer	IIIb
14	male	73	Gastric cancer	Ia
15	male	70	Gastric cancer	Ib
16	female	55	Gastric cancer	IIIa
17	male	62	Gastric cancer	IIIb
18	male	70	Gastric cancer	IV
19	female	51	Gastric cancer	IV
20	female	77	Gastric cancer	IV
21	female	84	Gastric cancer	IV
22	female	45	Gastric cancer	IIIc
23	male	83	Gastric cancer	IIIa
24	female	77	Rectum cancer	IIIb
25	female	67	Rectum cancer	IIa
26	male	74	Rectum cancer	IIb
27	male	82	Rectum cancer	IIa
28	female	75	Rectum cancer	IIa
