# Supplementary figures and images for: Expression of Four Autophagy-Related Genes Accurately Predicts the Prognosis of Gastrointestinal Cancer in Asian Patients
Source: Dis Markers. 2021 Aug 26;2021:7253633. doi: 10.1155/2021/7253633 (PMC8413069; doi:10.1155/2021/7253633)

TCGA-LIHC

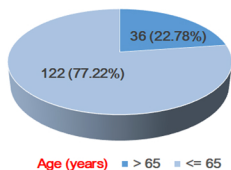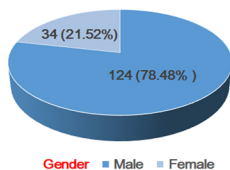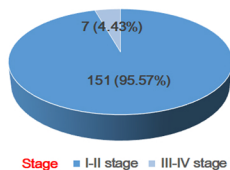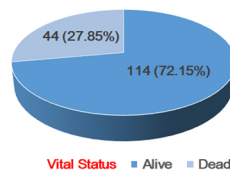

TCGA-STAD

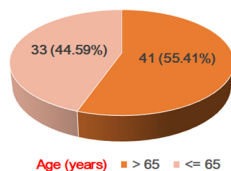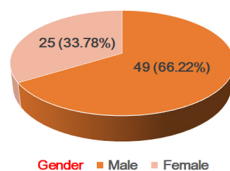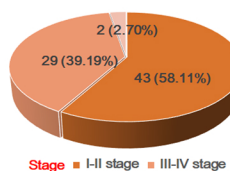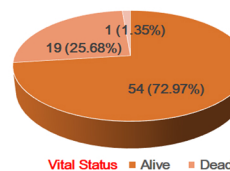

TCGA-ESCA

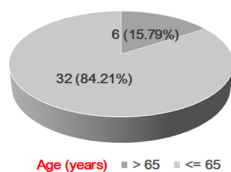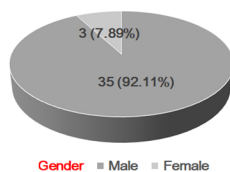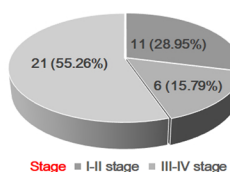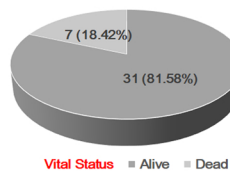

TCGA-PAAD

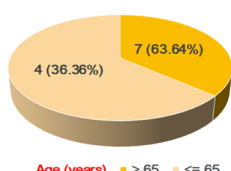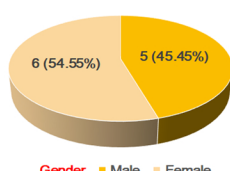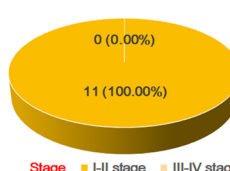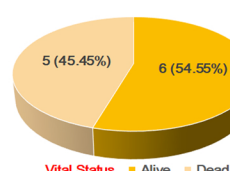

TCGA-COAD

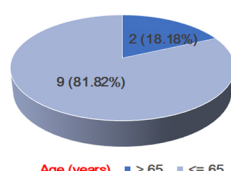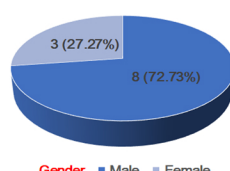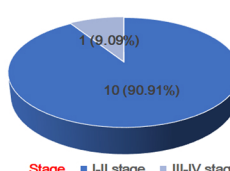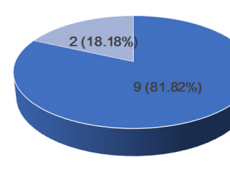

TCGA-CHOL

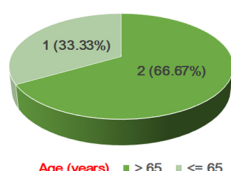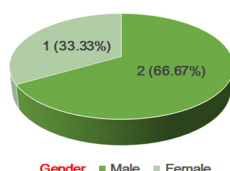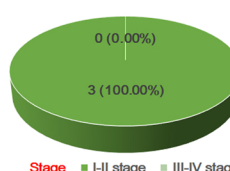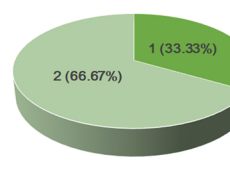

TCGA-READ

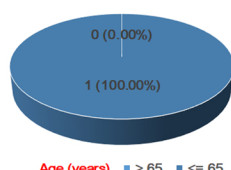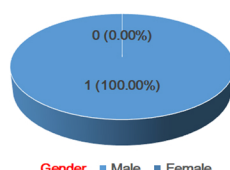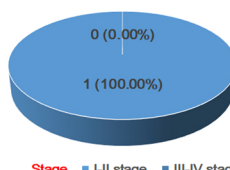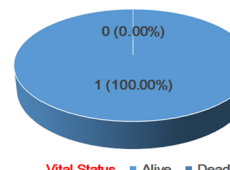

Supplement: Supplementary Materials — Table S1: the characteristics of tumor samples used in this study. Figure S1: clinical pathological parameters of Asian patients with gastrointestinal cancer in this research. Figure S2: GO and KEGG pathway enrichment analyses of the DEGs in GI cancers. (A) GO and (B) KEGG. Figure S3: prognosis-related ARGs based on LASSO regression analysis. (A) LASSO coefficient for the ARGs associated with the overall survival of GI cancer. (B) Plots of the cross-validation error rates. Figure S4: significant pathways in high- and low-risk groups of GI cancer patients: (A) GO based on GSEA; (B) KEGG based on GSEA. [file 7253633.f1.zip › 7253633.f1/Supplementary material Figure S1.pdf]

**A**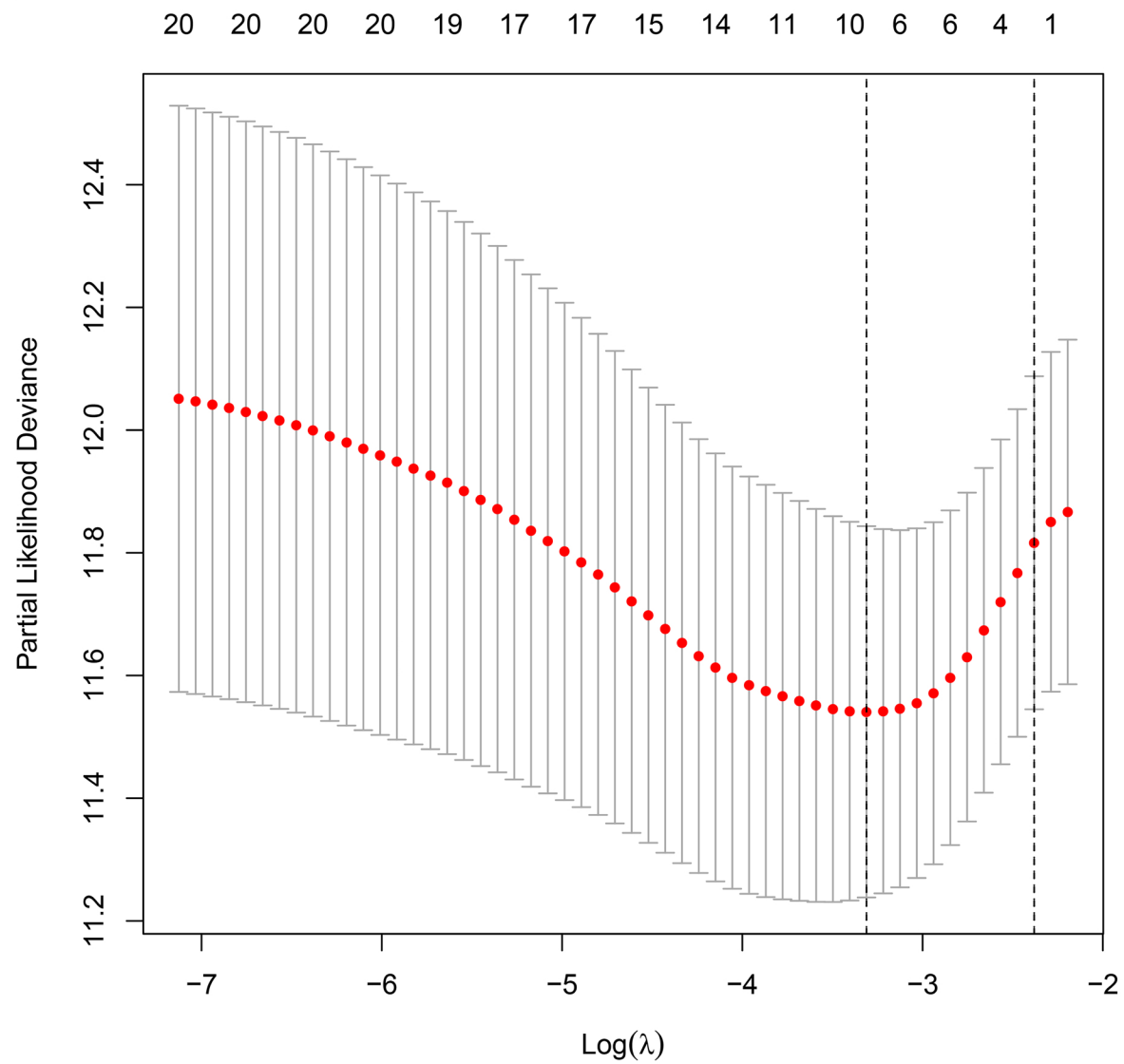**B**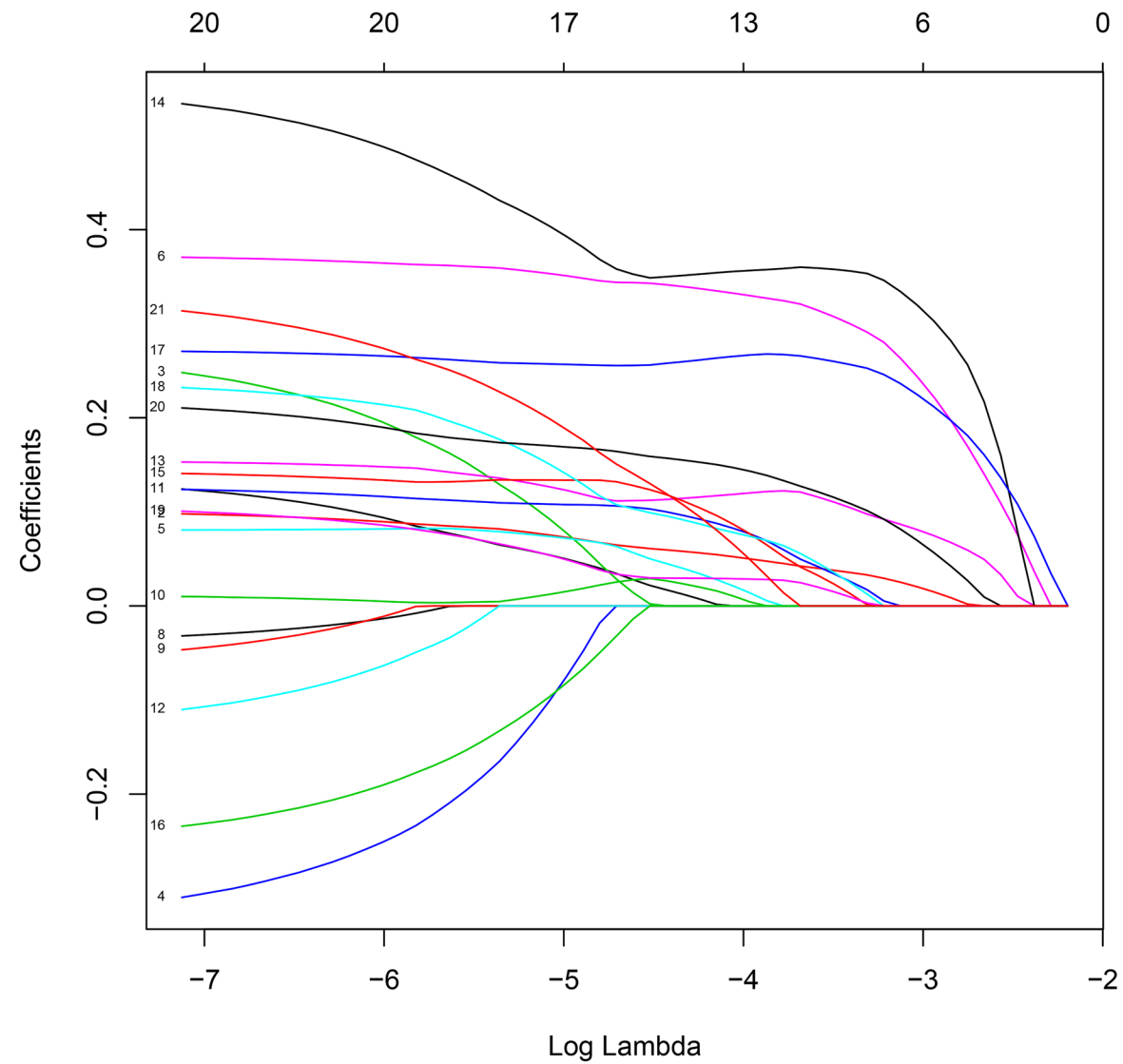

Supplement: Supplementary Materials — Table S1: the characteristics of tumor samples used in this study. Figure S1: clinical pathological parameters of Asian patients with gastrointestinal cancer in this research. Figure S2: GO and KEGG pathway enrichment analyses of the DEGs in GI cancers. (A) GO and (B) KEGG. Figure S3: prognosis-related ARGs based on LASSO regression analysis. (A) LASSO coefficient for the ARGs associated with the overall survival of GI cancer. (B) Plots of the cross-validation error rates. Figure S4: significant pathways in high- and low-risk groups of GI cancer patients: (A) GO based on GSEA; (B) KEGG based on GSEA. [file 7253633.f1.zip › 7253633.f1/Supplementary material Figure S3.pdf]

**A**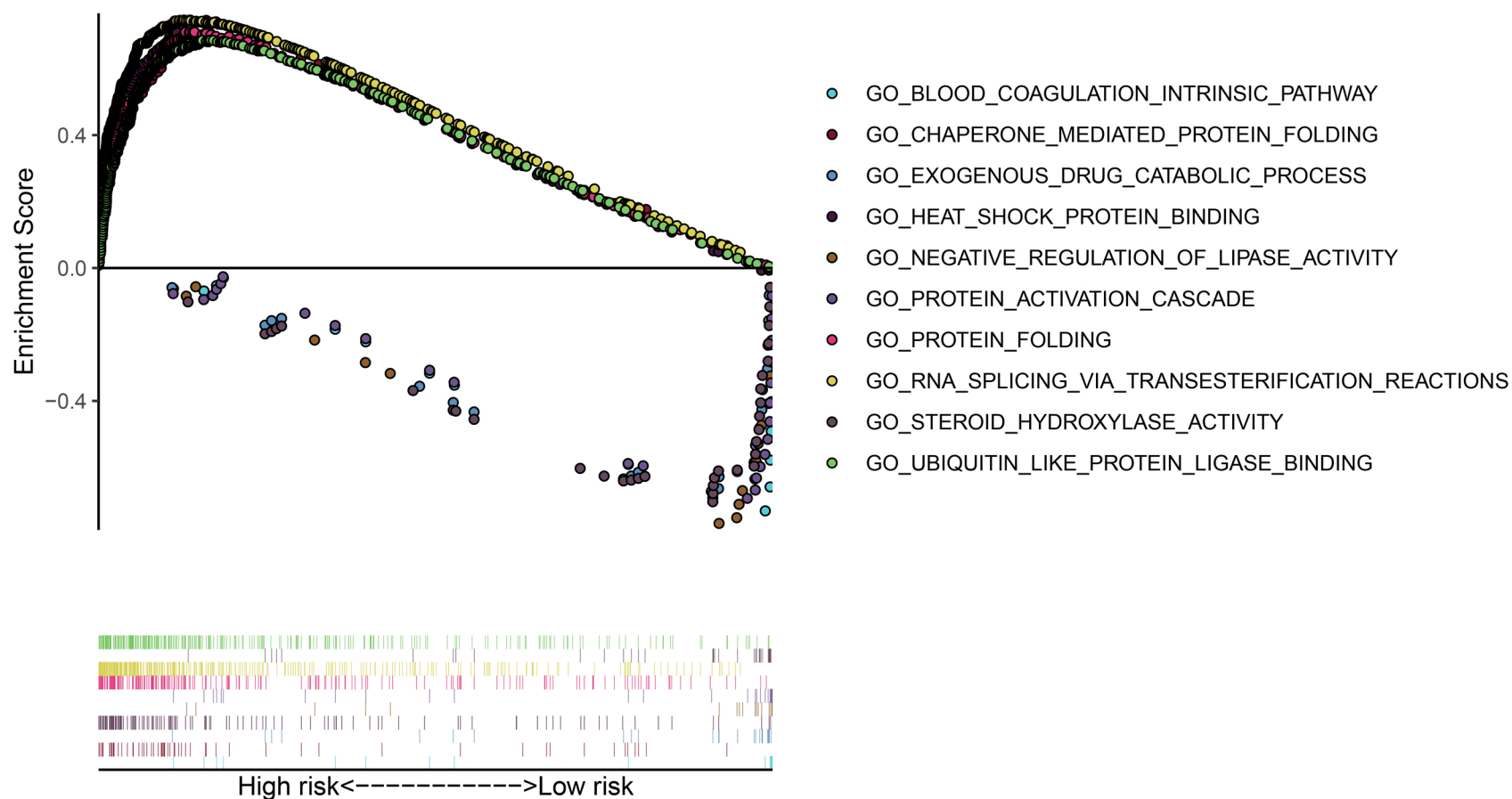**B**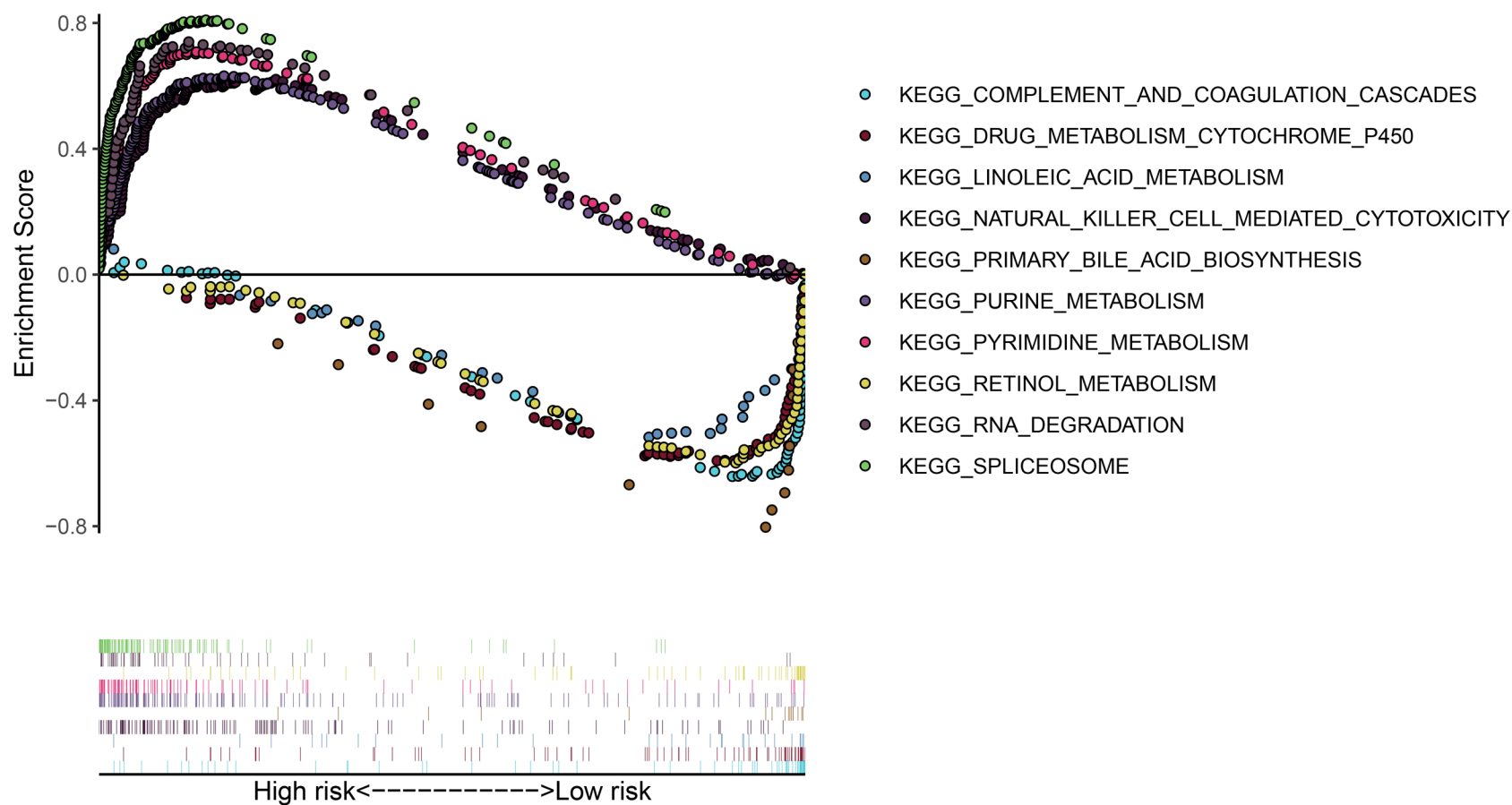

Supplement: Supplementary Materials — Table S1: the characteristics of tumor samples used in this study. Figure S1: clinical pathological parameters of Asian patients with gastrointestinal cancer in this research. Figure S2: GO and KEGG pathway enrichment analyses of the DEGs in GI cancers. (A) GO and (B) KEGG. Figure S3: prognosis-related ARGs based on LASSO regression analysis. (A) LASSO coefficient for the ARGs associated with the overall survival of GI cancer. (B) Plots of the cross-validation error rates. Figure S4: significant pathways in high- and low-risk groups of GI cancer patients: (A) GO based on GSEA; (B) KEGG based on GSEA. [file 7253633.f1.zip › 7253633.f1/Supplementary material Figure S4.pdf]
